# Comparative Study on Feature Extraction of Marine Background Noise Based on Nonlinear Dynamic Features

**DOI:** 10.3390/e25060845

**Published:** 2023-05-25

**Authors:** Guanni Ji, Yu Wang, Fei Wang

**Affiliations:** School of Zhongxing Communication, Xi’an Traffic Engineering Institute, Xi’an 710300, China; wangyulynne@sina.com (Y.W.); wf46863707@163.com (F.W.)

**Keywords:** marine background noise, feature extraction, nonlinear dynamics feature, entropy, Lempel–Ziv complexity

## Abstract

Marine background noise (MBN) is the background noise of the marine environment, which can be used to invert the parameters of the marine environment. However, due to the complexity of the marine environment, it is difficult to extract the features of the MBN. In this paper, we study the feature extraction method of MBN based on nonlinear dynamics features, where the nonlinear dynamical features include two main categories: entropy and Lempel–Ziv complexity (LZC). We have performed single feature and multiple feature comparative experiments on feature extraction based on entropy and LZC, respectively: for entropy-based feature extraction experiments, we compared feature extraction methods based on dispersion entropy (DE), permutation entropy (PE), fuzzy entropy (FE), and sample entropy (SE); for LZC-based feature extraction experiments, we compared feature extraction methods based on LZC, dispersion LZC (DLZC) and permutation LZC (PLZC), and dispersion entropy-based LZC (DELZC). The simulation experiments prove that all kinds of nonlinear dynamics features can effectively detect the change of time series complexity, and the actual experimental results show that regardless of the entropy-based feature extraction method or LZC-based feature extraction method, they both present better feature extraction performance for MBN.

## 1. Introduction

Marine background noise (MBN) is an eternal sound field in the marine environment that contains information about environmental characteristics such as water body, seabed, and sea surfaces [1,2]. For sonar with acoustic waves as the main means of detection and communication, it is necessary to consider the complex sound field with marine environmental noise as the background. Therefore, the study of ocean background noise, especially the study of feature extraction, is of great significance to the development of underwater acoustic weapons [3].

At present, traditional feature extraction methods mainly include frequency domain and time domain feature extraction methods [4,5,6], which can only effectively extract linear and stationary signals. However, MBN is a classic underwater acoustic signal with nonlinear, nonstationary, and non-Gaussian characteristics [7], and traditional feature extraction methods cannot effectively reflect its information [8,9]. While deep-learning-based methods also work well for feature extraction, they often require larger datasets and higher experimental configurations [10,11]. To address these shortcomings of the above methods, many scholars have studied a large number of nonlinear feature extraction methods, among which the mainstream methods are mainly based on two aspects of entropy and LZC [12,13]. This paper divides nonlinear dynamic features into two categories, entropy and Lempel–Ziv complexity (LZC), for comparative experimental analysis of MBN.

Entropy can be used to analyze signal complexity by virtue of its ability to characterize the degree of chaos in a time series [14]. Since the Shannon entropy theorem was put forward in 1948 [15], entropy has been widely used in various fields. In 1991, Pincus et al. first proposed approximate entropy (AE) [16], which improves the dependence of previous entropy on the length of time series and has a strong general ability. Sample entropy (SE) was first proposed by Richman et al. in 2000 [17]. Similar to AE, they are functions defined based on the unit step function, and SE can effectively reflect the information of signals with data loss. In 2007, Chen et al. proposed fuzzy entropy (FE) by combining the concepts of SE and fuzzy membership [18], which is an improved AE algorithm that reduces the signal loss of AE and SE due to the characteristics of unit step function during signal calculation. Unlike AE, SE, and FE, PE was proposed by Bandt et al. in 2002 [19], which is an improved entropy based on the Shannon entropy theorem; its calculation process is simple and has strong anti-noise ability. DE was proposed by Rostaghi et al. in 2016 [20]; it not only has the advantage of fast calculation speed, but also can reflect the amplitude change of signal, which is one of the most widely used entropies at present.

LZC is a significant theory in nonlinear dynamics, similar to entropy, and it is often used to evaluate the disorder and irregularity of signals [21]. The primary LZC algorithm was proposed by Lempel and Ziv in 1976 [22]. Due to the binary conversion of sequences, LZC has the advantages of no parameter setting and high computational efficiency, but the converted 0-1 sequence loses a lot of the original information of the sequence [23,24]. For this reason, Bai et al. [25] first combined LZC with entropy theory and proposed the permutation LZC (PLZC) by replacing the binary mapping with the permutation pattern in PE, which inherits the strong anti-noise ability of PE and improves the ability of LZC to characterize signals [26]. In 2020, Mao et al. [27] were inspired by the advantages of DE to effectively reflect amplitude information and integrated it into LZC to launch dispersion LZC (DLZC); the application of DLZC in various fields showed high stability and separability [28]. Dispersion entropy-based Lempel–Ziv complexity (DELZC) is a newly proposed complexity measure [29], which makes full use of the more effective dispersion pattern in DE to reflect more pattern information and further boosts the ability to capture the dynamic changes of signal.

The main contribution of this paper is the study of an MBN feature extraction method based on nonlinear dynamic features, where the entropy-based features include dispersion entropy (DE), permutation entropy (PE), fuzzy entropy (FE), and sample entropy (SE), and LZC-based features include LZC, dispersion LZC (DLZC), permutation LZC (PLZC), and dispersion entropy-based LZC (DELZC). Lastly, the separability of various types of features is compared by classification experiments of real MBNs. This paper is organized as follows: Section 2 provides a brief review of common entropy and LZC and conducts simulation experiments on their ability to detect time series complexity. In Section 3, we conducted the feature extraction method of MBN based on nonlinear dynamic features. Section 4 and Section 5 present the discussion and conclusions, respectively.

## 2. Nonlinear Dynamic Features

In this paper, nonlinear dynamic features are divided into two categories: entropy and Lempel–Ziv complexity (LZC). We introduce the relevant theories of entropy and LZC, respectively.

### 2.1. Entropy

**Figure 1:**
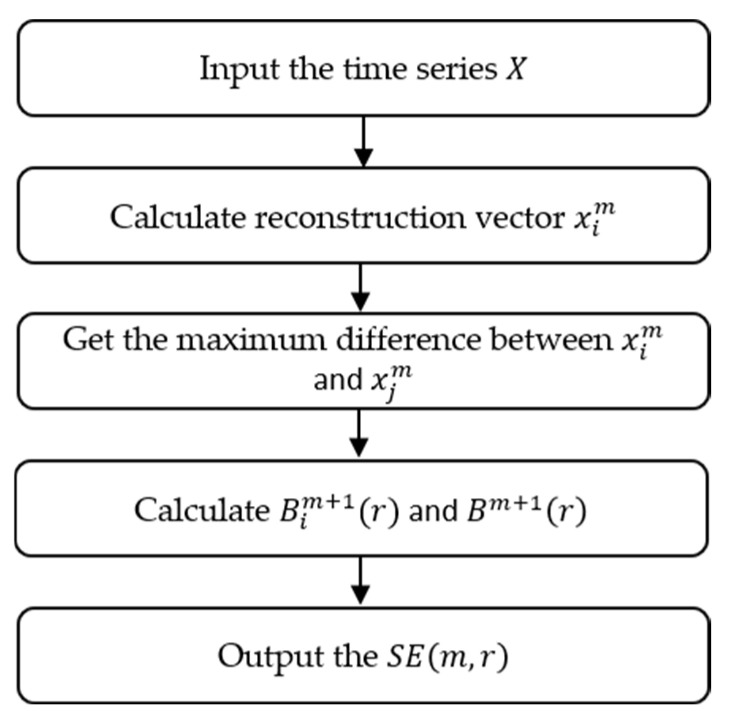
The calculation flow chart of *SE*.

Entropy can reflect the complexity of time series, among which SE, FE, PE, and DE are four common entropies, and their steps are explained in this section. The physical meanings of SE and FE are similar; these can measure the probability of the occurrence of new patterns in a time series. The specific steps of SE are as follows:

(1) For a specific time series $X=\left\{x_{i},\;i=1,\;2,\ldots ,n\right\}$, given an embedding dimension m, a set of vector sequences $\left\{x_{i}^{m},\;i=n-m+1\right\}$ can be obtained, where $x_{i}^{m}$ can be expressed as:
  (1) $$x_{i}^{m}=\left\{x_{i},\;x_{i+1},\ldots \;,\;x_{i+m-1}\right\}$$
(2) Define the absolute value of the maximum difference between the distance between vectors $x_{i}^{m}$ and $x_{j}^{m}$:
  (2) $$d=\left[x_{i}^{m},\;x_{j}^{m}\right]=\max\left|x_{i+k}-x_{j+k}\right|$$
  where k=0, 1, …, m−1.
(3) Given $x_{i}^{m}$, record the standard deviation of X as Std, count the number of j with d≤r as $B_{i}$, 0.1Std≤r≤0.25Std, and define $B_{i}^{m}\left(r\right)=\frac{1}{n-m-1}B_{i}$.
(4) $B^{m}\left(r\right)$ is defined as:
  (3) $$B^{m}\left(r\right)=\frac{1}{n-m}\overset{N-m}{\underset{i=1}{\sum }}B_{i}^{m}\left(r\right)$$
(5) Increase the embedding dimension to m+1, and repeat the above steps to obtain $B_{i}^{m+1}\left(r\right)$ and $B^{m+1}\left(r\right)$. The final expression of *SE* is:
  (4) $$SE\left(m,r\right)=\underset{n\to \infty }{\lim}\left\{\left.-\ln\left[\frac{B^{m+1}\left(r\right)}{B^{m}\left(r\right)}\right]\right\}\right.$$
  where the calculation flow chart of *SE* is shown in Figure 1.

*FE* introduces fuzzy membership degree $D^{m}\left(n,r\right)=e^{-\frac{d^{2}}{r}}$ based on *SE*, which can be expressed as:

(5) $$FE\left(m,r\right)=\underset{n\to \infty }{\lim}\left\{\left.-\ln\left[\frac{D^{m+1}\left(r\right)}{D^{m}\left(r\right)}\right]\right\}\right.$$

where m means embedding dimension, and 0.1Std≤r≤0.25Std.

The *PE* and *DE* are both developed based on Shannon entropy, where *PE* can be defined as follows:

(1) For the given time series $X=\left\{x_{e},\;e=1,\;2,\ldots ,n\right\}$, phase space reconstruction is performed to obtain Y:
  (6) $$Y=\left(\begin{array}{c} y_{1}\\ \begin{array}{c} \begin{array}{c} \vdots \\ y_{j} \end{array}\\ \vdots \end{array}\\ y_{K} \end{array}\right)$$
  (7) $$y_{j}=\left\{x_{j},\;x_{\left(j+t\right)},\;\ldots ,x_{\left(j+\left(m-1\right)t\right)}\;\right\}$$
  where m is the embedding dimension, t is the delay time, and $K=n-\left(m-1\right)t$.
(2) Reorder the elements in each reconstructed component in ascending order to obtain:
  (8) $$x_{\left(j+\left(i_{1}-1\right)t\right)}\leq x_{\left(j+\left(i_{2}-1\right)t\right)}\leq \ldots \;\leq x_{\left(j+\left(i_{m}-1\right)t\right)}$$

If $x_{\left(j+\left(i_{q1}-1\right)t\right)}=x_{\left(j+\left(i_{q2}-1\right)t\right)}$, then sort according to the size of i, that is, $x_{\left(j+\left(i_{q1}-1\right)t\right)}\leq x_{\left(j+\left(i_{q2}-1\right)t\right).}$

Finally, the new index of each group of elements is $S=\left\{i_{1},\;i_{2},\;\ldots ,i_{m}\right\}$, in which there are m! different time series, and the probability of each series occurrence are $P_{1},\;P_{2},\;\ldots P_{l}$.

**Figure 2:**
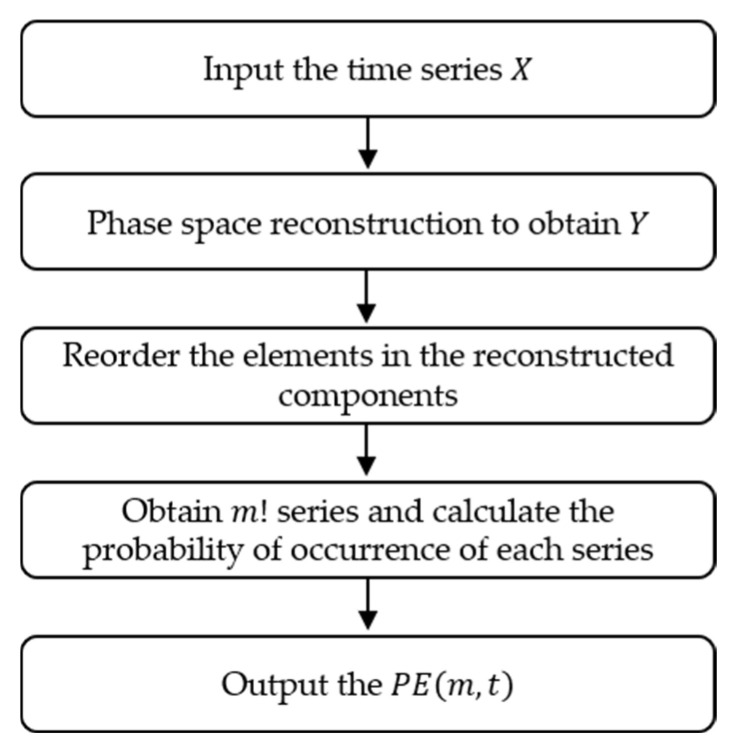
The calculation flow chart of *PE*.

(3) According to the Shannon entropy theorem, the expression of *PE* can be expressed as:
  (9) $$PE\left(m,t\right)=-\overset{l}{\underset{j=1}{\sum }}P_{j}lnP_{j}$$
  where Figure 2 displays the calculation flow chart of *PE*.

*DE* is an improved algorithm of *PE*, and its calculation formula is:

(10) $$DE\left(m,c,\;t\right)=-\overset{c^{m}}{\underset{u=1}{\sum }}P_{u}lnP_{u}$$

where m signifies the embedding dimension, c represents the number of categories, and t is the delay time.

### 2.2. Lempel–Ziv Complexity

Lempel–Ziv complexity is an important branch of nonlinear dynamics, among which LZC, PLZC, DLZC, and DELZC are the most representative ones. This section gives the calculation steps of these four LZC-based features.

**Figure 3:**
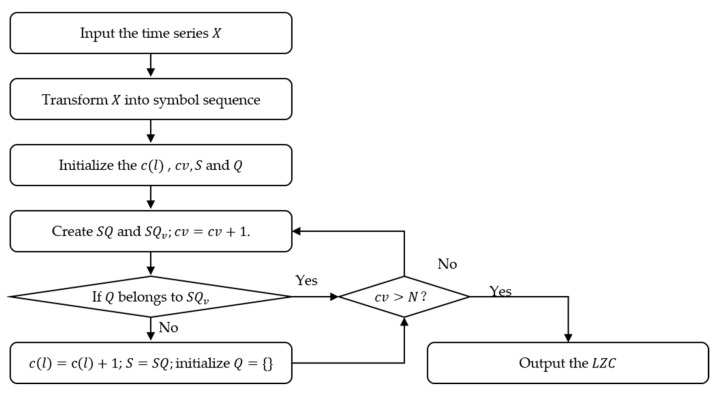
The calculation flow chart of *LZC*.

LZC is the primitive algorithm, which reflects the complexity of time series by counting the occurrence rate of new patterns in the sequence. The calculation flow chart of LZC is illustrated in Figure 3, and specific steps are as follows:

(1) For time series $X=\left\{x_{i},i=1,2,3,\ldots ,N\right\}$, each element is converted to 0 or 1 by the following formula:
  (11) $$y_{i}=\left\{\begin{array}{c} 0,\;\;\;\;\;\;\;\;\;\;\;if\;x_{i}<\bar{x}\\ 1,\;\;\;\;\;\;\;\;otherwise \end{array}\right.$$
  where $\bar{x}$ is the mean value of sequence X, then the symbol sequence $Y=\{y_{i},i=1,2\ldots \ldots ,N\}$ is obtained.
(2) Initialize the complexity index $c\left(l\right)\;$ and count value cv to 0 and 1, respectively, and let S and Q denote the first and second elements in Y. By merging S  and Q into SQ, $SQ_{v}$ is obtained by removing the last element of SQ.
(3) Judge whether Q belongs to $SQ_{v}$. If so, update Q by adding the next character. Otherwise, $c\left(l\right)=c\left(l\right)+1$, S=SQ, and initialize Q={}. For each judgment that is performed, the updated SQ and updated $SQ_{v}\;$ are obtained in the same way as Step (2), and cv=cv+1.
(4) Judge whether cv exceeds l; if not, return to Step (3); otherwise, the calculation of complexity is completed.
(5) The normalized result of *LZC* can be expressed as:
  (12) $$LZC=\frac{c\left(l\right)\log_{2}l}{l}$$

**Figure 4:**
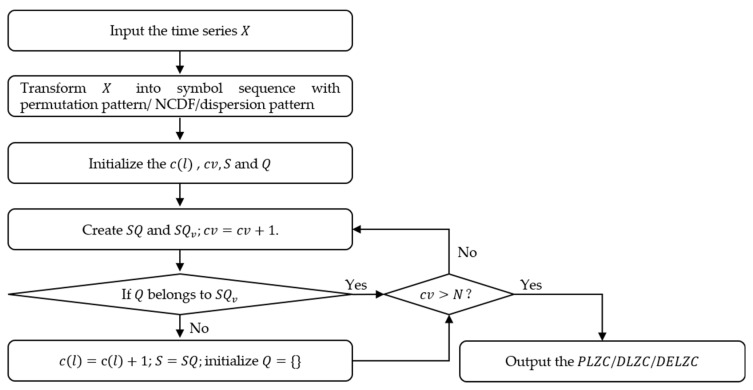
The calculation flow chart of *PLZC*, *DLZC*, and *DELZC*.

*PLZC*, *DLZC*, and *DELZC* are presented by improving the mapping of the original sequence in *LZC* Step (1). *PLZC* uses the permutation pattern in *PE* to generate the symbol sequence for *LZC*; *DLZC* and *DELZC* increase the number of categories in the symbol sequence by referring to different steps in *DE*. The calculation flow chart of *PLZC*, *DLZC*, and *DELZC* is shown in Figure 4.

For *PLZC*, the calculation process includes Step (1) and Step (2) of *PE* in Section 2.1, then we name the obtained permutation pattern according to the corresponding pattern category to obtain the symbol sequence; finally, the value of *PLZC* is obtained according to *LZC* Step (2) to Step (5). It is noteworthy that the calculation formula will also change as the number of element categories in the symbol sequence increases, and the specific formula is as follows:

(13) $$PLZC=\frac{c\left(l\right)\log_{m!}l}{l}$$

where m is the embedding dimension.

For *DLZC* and *DELZC*, these two algorithms are proposed by introducing the normal cumulative distribution function (NCDF) and dispersion pattern in *DE* into the original *LZC*, respectively. *DLZC* employs NCDF and a rounding function to convert the original sequence into a symbol sequence with c categories; in *DELZC*, after the conversion of NCDF and rounding function, the phase space is reconstructed to obtain a variety of dispersion patterns, and then the symbol sequence is obtained in a similar way to *PLZC*. Through the above processing, the calculation formulas of *DLZC* and *DELZC* are as follows:

(14) $$DLZC=\frac{c\left(l\right)\log_{c}l}{l}$$

(15) $$DELZC=\frac{c\left(l\right)\log_{c^{m}}l}{l}$$

where c is the number of categories and m is the embedding dimension.

### 2.3. Simulation Experiment Verification

For the nonlinear dynamics characterized in the previous section, the MIX signal is introduced as a reflection of their ability to detect changes in the degree of chaos of the time series. The MIX signal consists of a periodic signal X1, a random signal X2, and a controlling parameter u. By artificially changing the parameter u, we can control the randomness of the entire synthesized signal. The MIX signal can be defined as follows:

(16) $$\left\{\begin{array}{c} \mathrm{MIX}=\left(1-u\right)\times X1+u\times X2\\ X1=\sqrt{2}sin\frac{2\pi t}{12}\\ X2\in \left[-\sqrt{3},\sqrt{3}\right] \end{array}\right.$$

**Figure 5:**
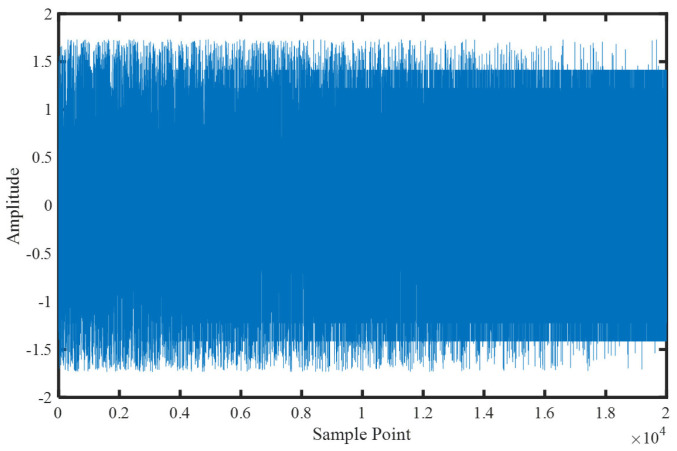
Time domain waveform of the MIX signal.

In the comparative experiments of this subsection, u is linearly decreased from an initial value of 0.99 to a final value of 0.01. The sampling frequency is 1000 Hz, and the total length is 20 s. The time domain waveform of the MIX signal is shown in Figure 5, where it can be visually observed that the signal becomes increasingly stable. In this section, sliding windows with a length of 1 s and 90% overlap are used to extract sample signals, resulting in a total of 190 segments. By calculating various entropy values for each segment, the ability of each type of entropy to detect changes in the chaos of the time series is examined.

**Figure 6:**
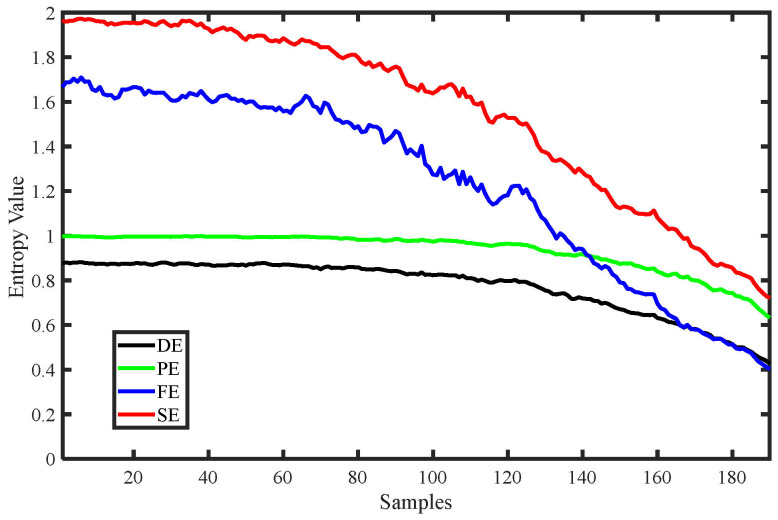
Various entropy change curves of MIX signal.

Various entropy change curves of the MIX signal are shown in Figure 6, including DE, PE, SE, and FE, and Table 1 shows the parameter settings of these four entropies. From Figure 6, it can be seen that all the entropy value curves generally decrease as the complexity of the MIX signal decreases, indicating that various entropies can reflect changes in the degree of chaos in the time series. Among these, the curves of DE and PE are relatively stable, indicating strong stability of the entropy values, while FE and SE exhibit larger fluctuations but are able to more clearly reflect changes in the degree of chaos in the signal during the early stages. In conclusion, all four entropies can effectively reflect changes in the degree of chaos in the time series.

**Figure 7:**
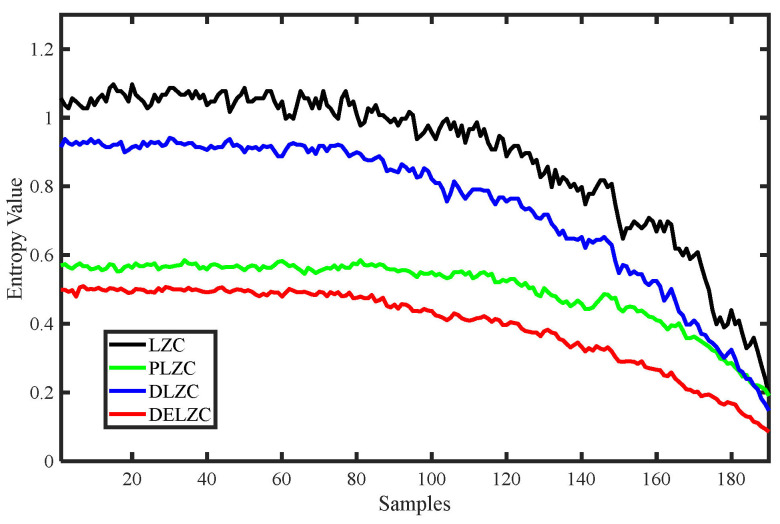
Various complexity change curves of MIX signal.

Similarly, we conducted the same experiments for four types of LZCs, including LZC, PLZC, DLZC, and DELZC; the obtained complexity value curves are shown in Figure 7, and their parameters are also shown in Table 2. From Figure 7, it can be observed that as the complexity of the MIX signal continuously decreases, the change curves of all four LZCs also show a decreasing trend, while the remaining three complexities except LZC also show a strong stability in characterizing the degree of MIX signal confusion. Therefore, it can be concluded that all four complexities can also effectively reflect the change of the chaos degree of the time series.

## 3. Feature Extraction of MBN Based on Nonlinear Dynamic Features

### 3.1. Marine Background Noise

**Figure 8:**
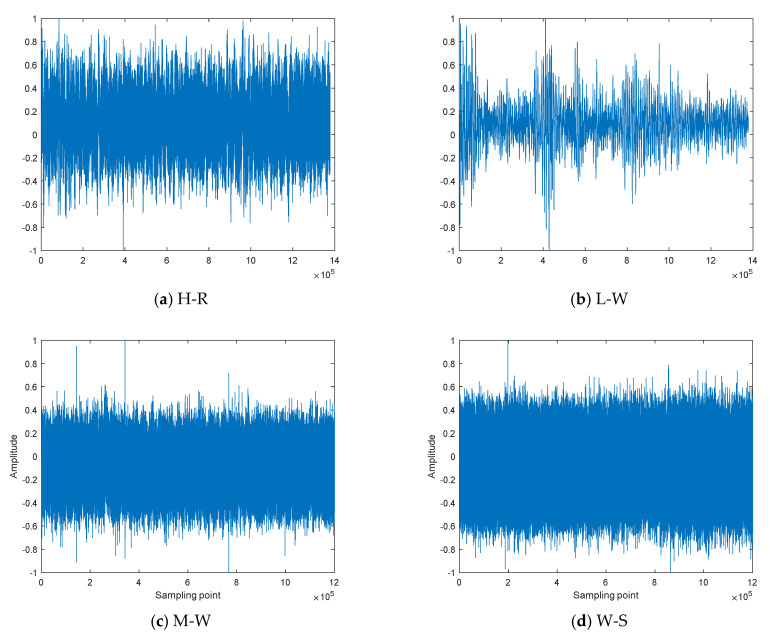
The normalized MBN.

Four types of measured MBN are selected from the dataset of the National Marine Park Service [30] to study and compare the feature extraction method based on nonlinear dynamic features, including heavy rain on the sea surface, light wind on the sea surface, moderate wind on the sea surface, and wind and ship noise on underwater hydrophones, named H-R, L-W, M-W, and W-S, respectively. For each type of MBN, 100 samples are randomly selected, where each sample contains 4096 sampling points, and the normalized MBN is shown in Figure 8.

### 3.2. Feature Extraction and Analysis Based on Entropy

In this experiment, four common entropies including DE, PE, FE, and SE are selected for feature extraction experiments of MBN.

#### 3.2.1. Parameter Setting of Entropy Features

To effectively compare the effect of four entropies on feature extraction for MBN, we selected the parameters when DE, PE, FE, and SE have the best effect on feature extraction for four MBN, in which the parameters of DE and PE are m=4, t=1, respectively, and the parameter of DE is c=6; the parameters of FE and SE are set to r=0.25, and m is 4 and 1, respectively. The specific details are the same as in Table 1.

#### 3.2.2. Single Feature Extraction and Classification

**Figure 9:**
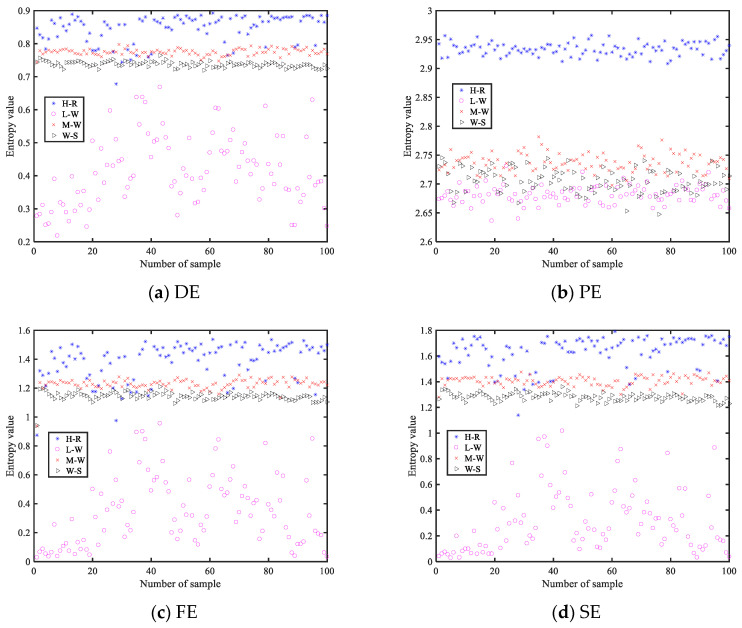
The feature distribution of the four entropies for MBN.

To verify the advantages and disadvantages of the four entropies, feature extraction methods based on DE, PE, FE, and SE are used to carry out single feature extraction experiments for four MBN. Figure 9 displays the feature distribution of the four entropies for MBN.

As you can see in Figure 9, the four entropies can distinguish at least one MBN, and M-W and W-S are the most difficult to identify for all sub-figures; for DE, its ordinate range is the smallest, and of the feature values of the four MBN, it is the densest; from the feature distribution figure of PE, it can be seen that the entropy values of three MBN are mixed together, which is the most overlapping part of all sub-figures; for FE and SE, their differentiation effect on the four MBN is almost the same, and the overlap of the four MBN similar to DE is less. It can be concluded that among all feature extraction methods, the feature extraction method based on DE has the best effect for four MBN, and the feature extraction method based on PE is the worst.

**Figure 10:**
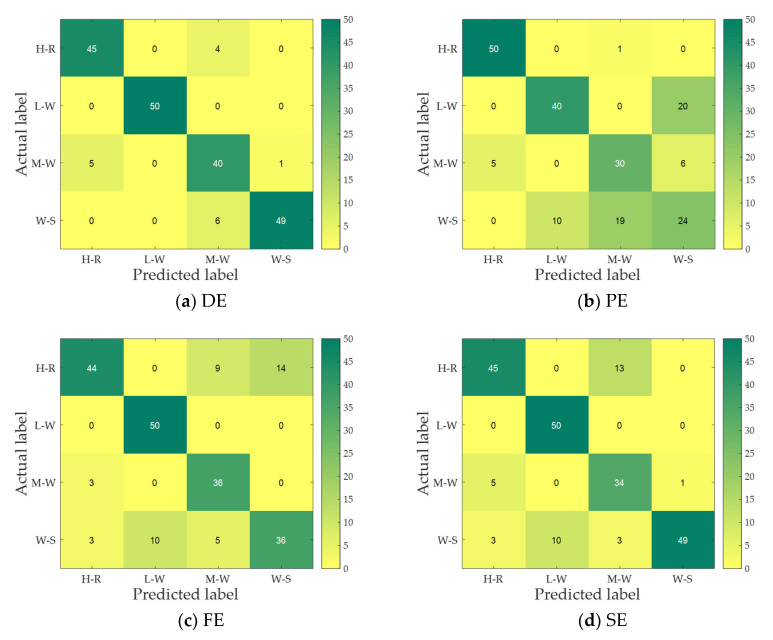
The confusion matrix of four entropies for MBN.

To compare the recognition results of each entropy more easily for four MBN, a K-nearest neighbor (KNN) classifier is applied to classify and identify four MBN. For each entropy, 50 samples of each MBN are randomly selected as training samples, and then the remaining 50 samples are used as test samples. Figure 10 presents the confusion matrix of four entropies for MBN, and we further calculate the recognition rate of four entropies to MBN. Table 3 shows the recognition rate of four entropies.

As can be seen from Figure 10 and Table 3, corresponding to the feature distribution figure, each entropy has a recognition rate of 100% for one MBN; L-W is the easiest to recognize, and only 10 samples are identified incorrectly for all entropy indexes; for PE, except for the H-R, all MBN have the largest number of false identification samples, and W-S is the most difficult to identify; for all confusion matrix figures, only the number of samples for correct identification of each signal in Figure 10a is not less than 40; Table 2 shows that DE has the highest recognition rate for the four MBN and PE has the lowest recognition rate. In short, compared with other feature extraction methods, the feature extraction method based on DE has the best recognition effect for four MBN.

#### 3.2.3. Multiple Feature Extraction and Classification

Although single feature extraction has achieved good results, it still cannot fully identify the different types of MBN. To further improve the feature extraction effect for MBN, we perform multiple feature extraction, extracting two, three, and four features, respectively. The highest recognition rates of four entropies under multiple feature extraction are listed in Table 4.

As seen from Table 4, multiple feature extraction methods significantly improve the recognition rate of single feature extraction methods, and the highest recognition rate of 97.5% is achieved when two or three features are extracted, which is 6% higher than the highest recognition rate for the single feature extraction methods. Moreover, in multiple feature extraction, regardless of how many features are extracted, the selected features all contain DE. However, the recognition rate does not always increase as the number of extracted features increases. When four features are extracted, the recognition rate decreases instead.

### 3.3. Feature Extraction and Analysis Based on LZC

In this section, we select another kind of nonlinear dynamic feature to extract the features of MBN and analyze them, including LZC, PLZC, DLZC, and DELZC.

#### 3.3.1. Parameter Setting of LZC-Based Features

For the purpose of comparing the performance of four LZC-based features in MBN feature extraction, we set the common parameters of these features to be consistent. Among them, LZC does not need parameter settings; the time delay τ and the embedding dimension m  of PLZC and DELZC are set to 1 and 4, respectively; the number of categories c of DELZC and DLZC is uniformly set to 6, and the specific details are the same as in Table 2.

#### 3.3.2. Feature Extraction and Classification

**Figure 11:**
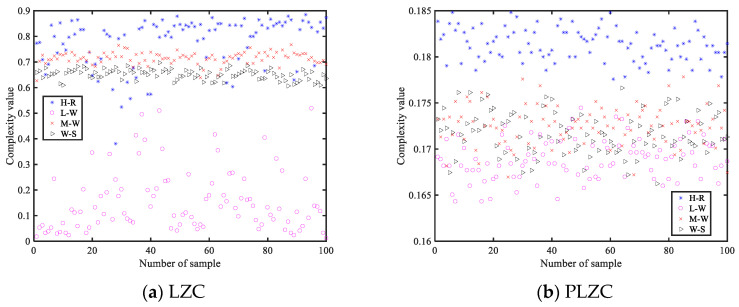

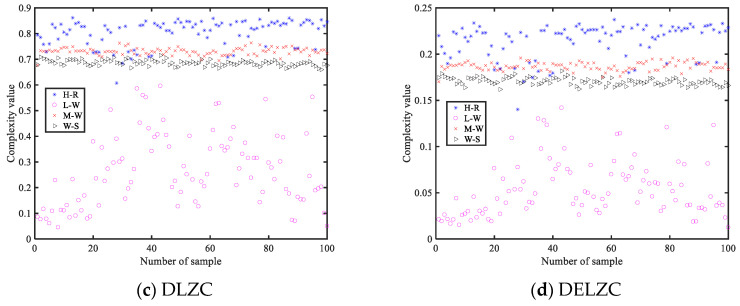
The feature distributions of four LZC-based features for MBN.

To intuitively show the feature extraction effect of four LZC-based features on different MBN, this section gives the feature distributions of each LZC-based feature. Figure 11 shows the feature distributions of four LZC-based features for MBN.

According to the observations in Figure 11, it can be seen that for the four LZC features, the distribution of the L-W signal samples is the messiest and accompanied by large fluctuation; for PLZC, the aliasing phenomenon of feature distribution is the most serious, and the samples of L-W, M-W, and W-S almost completely overlap; compared with LZC, DLZC and DELZ can better distinguish H-R from the other three signals due to fewer overlapping samples. To sum up, DLZC and DELZC have stronger recognition ability for four MBN.

**Figure 12:**
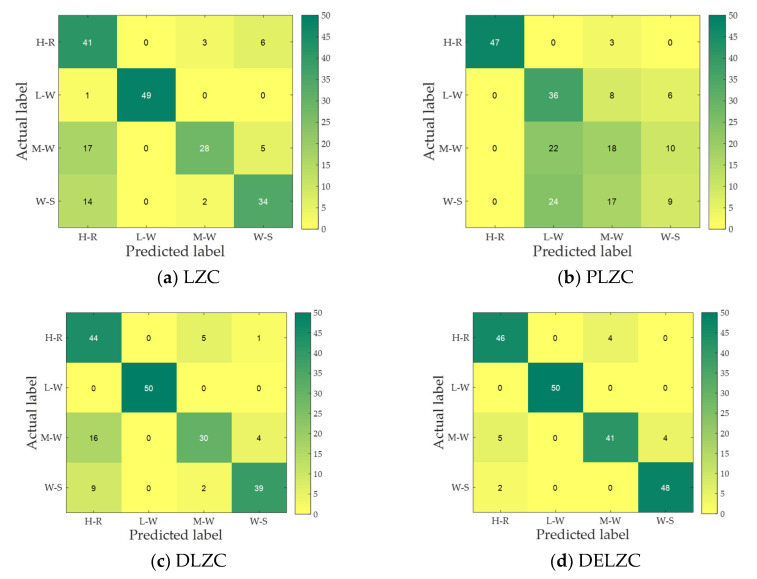
The confusion matrix of four LZC-based features for MBN.

From the Figure 11 and the above analysis, it is obvious that only relying on the feature distribution cannot determine which LZC-based features perform best in feature extraction. For this reason, we used a KNN classifier to classify different types of MBN, and the number of misclassified samples and recognition rate are used as the criteria for evaluating the effect of each LZC-based feature. Figure 12 and Table 5 illustrate the confusion matrix of four MBN and the recognition rate of four MBN, respectively.

From Figure 12 and Table 5, it can be concluded that different LZC-based features have different recognition effects on various MBN; of all LZC-based features, PLZC has the most misclassified samples and the lowest average recognition rate for the four MBN; for DELZC, its distinguishing effect on the four MBN is significantly better than LZC, PLZC, and DLZC, and the average recognition rate is at least 11% higher than the other three features. On the whole, the recognition result conforms to the situation shown by the feature distribution, and it can be concluded from the recognition result that DELZC has the most outstanding performance in feature extraction of four MBN.

#### 3.3.3. Multiple Feature Extraction and Classification

As with entropy, to further improve the performance of feature extraction, we also carry out multiple feature extraction experiments and extracted two, three, and four features, respectively. Table 6 presents the highest recognition rate of four LZC-based features under multiple feature extraction.

From Table 6, the highest recognition rate is achieved when two features or four features are extracted, reaching 95.5%, which is significantly higher than the highest recognition rate of single feature extraction methods. As with entropy-based multiple feature extraction experiments, it is not the case that the higher the number of features extracted, the higher the recognition rate.

## 4. Discussion

**Figure 13:**
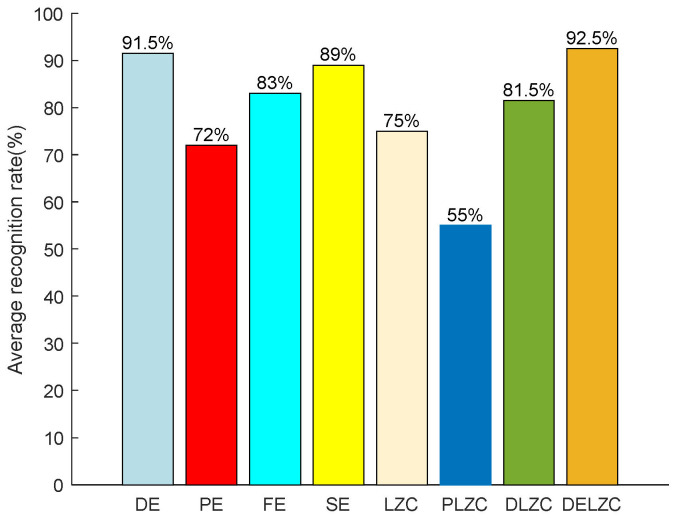
The average recognition rate of feature extraction methods based on eight nonlinear dynamic features for MBN.

In this paper, we carry out the experiments of MBN feature extraction based on entropy and LZC in the experimental part, in which the entropy of comparison includes DE, PE, FE, and SE, and the LZC of comparison includes LZC, PLZC, DLZC, and DELZC. Finally, the classification algorithm KNN is used to calculate recognition effects. In future research, we will use new deep-learning-based methods for classification and recognition [31,32]. To further compare the effect of different nonlinear dynamic features on feature extraction, Figure 13 shows the average recognition rate of feature extraction methods based on eight nonlinear dynamic features for MBN.

It can be seen from Figure 13 that DELZC has the highest recognition rate of 92.5%, and PLZC has the lowest recognition rate of 55%; for the entropy-based feature extraction method, the recognition rate is higher than 70%, and DE has the highest average recognition rate; in addition, for the LZC-based feature extraction method, except for PLZC, the recognition rate based on other features is higher than 75%; last but not least, regardless of entropy-based feature extraction method or LZC-based feature extraction method, they both have their own advantages, and both show better feature extraction performance for marine background noise signals.

In addition, to further explore the effect of multiple feature extraction, we also conduct hybrid multiple feature extraction, i.e., mixing entropy and LZC together as the subjects of feature extraction at the same time. Table 2 demonstrates the highest recognition rate for hybrid multiple feature extraction.

It is clear from Table 7 that hybrid multiple feature extraction is significantly more effective than extracting only entropy or LZC, and the highest recognition rate can reach 98% when the number of extracted features is between two and six. As with the entropy-based and LZC-based multiple feature extraction experiments, the recognition rate does not always increase as the number of extracted features increases. The recognition rate stays the same at first, but eventually the recognition rate drops instead. The more features extracted, the higher the recognition rate, but it is not the case that the more features, the better. When a few features can obtain the highest accuracy, the more features are selected, the more redundant they are, resulting in a decrease in recognition rate. Therefore, there will be a phenomenon where the more features extracted, the lower the recognition rate.

## 5. Conclusions

This paper studies the feature extraction method of MBN based on nonlinear dynamic features, especially the feature extraction methods based on entropy or LZC and compares the different feature extraction methods through measured MBN. The main conclusions are as follows: (1) for entropy-based MBN single feature extraction methods, the feature extraction method based on dispersion entropy has the highest recognition rate of 91.5%, which is 19.5%, 8.5%, and 2.5% higher than the recognition rates of PE, FE, and SE, respectively; (2) for LZC-based MBN single feature extraction methods, the feature extraction method based on DELZC has the highest recognition rate of 92.5%, which is 17.5%, 37.5%, and 11% higher than the recognition rates of LZC, PLZC, and DELZC, respectively; (3) whether for entropy-based multiple feature extraction method or LZC-based multiple feature extraction method, they both significantly improve the recognition rate of single feature extraction methods; and (4) it is not the case that the higher the number of features extracted, the higher the recognition rate, and as the number of features continues to increase, the recognition rate may remain the same or even decrease.

## Figures and Tables

**Table 1:** The parameter settings of four entropies.

Feature	Parameter			
	m	c	r	t
DE	4	6	−	1
PE	4	−	−	1
FE	4	−	0.25	−
SE	1	−	0.25	−

**Table 2:** The parameter settings of four LZCs.

Feature	Parameter		
	m	τ	c
LZC	−	−	−
PLZC	4	1	−
DLZC	−	−	6
DELZC	4	1	6

**Table 3:** The recognition rate of four entropies for MBN.

Feature	Category of Signal				Average Recognition Rate
	H-R	L-W	M-W	W-S	
DE	88.0%	100.0%	80.0%	98.0%	91.5%
PE	100.0%	80.0%	60.0%	48.0%	72.0%
FE	88.0%	100.0%	72.0%	72.0%	83.0%
SE	90.0%	100.0%	68.0%	98.0%	89.0%

**Table 4:** The highest recognition rates of four entropies under multiple feature extraction.

	Number of Extracted Features		
	Two	Three	Four
Highest recognition rate	97.5%	97.5%	96.5%
Selected features	DE, PE	DE, FE, SE	All features

**Table 5:** The recognition rate of four LZC-based features for MBN.

Feature	Category of Signal				Average Recognition Rate
	H-R	L-W	M-W	W-S	
LZC	82.0%	98.0%	56.0%	64.0%	75.0%
PLZC	94.0%	72.0%	36.0%	18.0%	55.0%
DLZC	88.0%	100%	60.0%	78.0%	81.5%
DELZC	92.0%	100%	82.0%	96.0%	92.5%

**Table 6:** The highest recognition rate of four LZC-based features under multiple feature extraction.

	Number of Extracted Features		
	Two	Three	Four
Highest recognition rate	95.5%	95.0%	95.5%
Selected features	LZC, DELZC	LZC, PLZC, DLZC	All features

**Table 7:** The highest recognition rate for hybrid multiple feature extraction.

	Number of Extracted Features						
	Two	Three	Four	Five	Six	Seven	Eight
Highest recognition rate	98.0%	98.0%	98.0%	98.0%	98.0%	97.5%	96.0%

## Data Availability

The data used to support the findings of this study are available from the corresponding author upon request.
